# Renal osteodystrophy and clinical outcomes: data from the Brazilian Registry of Bone Biopsies - REBRABO

**DOI:** 10.1590/2175-8239-JBN-2019-0045

**Published:** 2020-01-20

**Authors:** Cinthia Esbrile Moraes Carbonara, Luciene Machado dos Reis, Kélcia Rosana da Silva Quadros, Noemi Angelica Vieira Roza, Rafael Sano, Aluizio Barbosa Carvalho, Vanda Jorgetti, Rodrigo Bueno de Oliveira

**Affiliations:** 1 Universidade Estadual de Campinas, Faculdade de Ciências Médicas, Laboratório para o Estudo do Distúrbio Mineral e Ósseo em Nefrologia, Campinas, SP, Brasil.; 2 Universidade Estadual de Campinas, Faculdade de Ciências Médicas, Campinas, SP, Brasil.; 3 Universidade de São Paulo, Faculdade de Medicina, Hospital das Clínicas, Laboratório de Fisiopatologia Renal, São Paulo, SP, Brasil.; 4 Sociedade Brasileira de Nefrologia, Departamento de Distúrbios do Metabolismo Ósseo Mineral na Doença Renal Crônica, São Paulo, SP, Brasil.; 5 Universidade Federal de São Paulo, São Paulo, SP, Brasil.

**Keywords:** Renal Insufficiency, Chronic, Chronic Kidney Disease-Mineral and Bone Disorder, Aluminum accumulation, Osteoporosis, Treatment Outcome, Insuficiência Renal Crônica, Distúrbio Mineral e Ósseo na Doença Renal Crônica, Osteoporose, Resultado do Tratamento

## Abstract

**Introduction:**

Mineral and bone disorders (MBD) are major complications of chronic kidney disease (CKD)-related adverse outcomes. The Brazilian Registry of Bone Biopsy (REBRABO) is an electronic database that includes renal osteodystrophy (RO) data. We aimed to describe the epidemiological profile of RO in a sample of CKD-MBD Brazilian patients and understand its relationship with outcomes.

**Methods:**

Between August 2015 and March 2018, 260 CKD-MBD stage 3-5D patients who underwent bone biopsy were followed for 12 to 30 months. Clinical-demographic, laboratory, and histological data were analyzed. Bone fractures, hospitalizations, and death were considered the primary outcomes.

**Results:**

Osteitis fibrosa, mixed uremic osteodystrophy, adynamic bone disease, osteomalacia, osteoporosis, and aluminum (Al) accumulation were detected in 85, 43, 27, 10, 77, and 65 patients, respectively. The logistic regression showed that dialysis vintage was an independent predictor of osteoporosis (OR: 1.005; CI: 1.001-1.010; *p =* 0.01). The multivariate logistic regression revealed that hemodialysis treatment (OR: 11.24; CI: 1.227-100; *p =* 0.03), previous parathyroidectomy (OR: 4.97; CI: 1.422-17.241; *p =* 0.01), and female gender (OR: 2.88; CI: 1.080-7.679; *p =* 0.03) were independent predictors of Al accumulation; 115 patients were followed for 21 ± 5 months. There were 56 hospitalizations, 14 deaths, and 7 fractures during follow-up. The COX regression revealed that none of the variable related to the RO/turnover, mineralization and volume (TMV) classification was an independent predictor of the outcomes.

**Conclusion:**

Hospitalization or death was not influenced by the type of RO, Al accumulation, or TMV classification. An elevated prevalence of osteoporosis and Al accumulation was detected.

## RESUMO

**Introdução:**

Os distúrbios minerais e ósseos (DMO) são importantes complicações da doença renal crônica (DRC) associadas à desfechos adversos. O Registro Brasileiro de Biópsia Óssea (REBRABO) é um banco de dados eletrônico que inclui dados sobre osteodistrofia renal (OR). Nosso objetivo foi descrever o perfil epidemiológico da OR em uma amostra de pacientes brasileiros com DMO-DRC e entender sua associação com os desfechos.

**Métodos:**

Entre agosto de 2015 e março de 2018, 260 pacientes com DMO-DRC estágio 3-5D submetidos à biópsia óssea foram acompanhados por 12 a 30 meses. Dados clínico-demográficos, laboratoriais e histológicos foram analisados. Fraturas ósseas, hospitalizações e óbito foram considerados como desfechos primários.

**Resultados:**

Osteíte fibrosa, osteodistrofia urêmica mista, doença óssea adinâmica, osteomalácia, osteoporose e acúmulo de alumínio (Al) foram detectados em 85, 43, 27, 10, 77 e 65 pacientes, respectivamente. A regressão logística mostrou que o tempo em diálise foi um preditor independente de osteoporose (OR: 1.005; IC: 1.001-1.010; *p =* 0,01). A regressão logística multivariada revelou que o tratamento hemodialítico (OR: 11,24; IC: 1,227-100; *p =* 0,03), paratireoidectomia prévia (OR: 4,97; IC: 1,422-17,241; *p =* 0,01) e sexo feminino (OR: 2,88; IC: 1,080-7,679; *p =* 0,03) foram preditores independentes de acúmulo de Al; 115 pacientes foram acompanhados por 21 ± 5 meses. Houve 56 internações, 14 óbitos e 7 fraturas durante o seguimento. A regressão COX revelou que nenhuma das variáveis relacionadas ao tipo de OR/remodelação-mineralização-volume (classificação TMV) foi um preditor independente de desfechos.

**Conclusão:**

A hospitalização ou óbito não foram influenciadas pelo tipo de OR, acúmulo de Al ou classificação de TMV. Foi detectada uma prevalência elevada de osteoporose e acúmulo de Al.

## INTRODUCTION

Chronic kidney disease (CKD) is a worldwide public health problem with an increasing prevalence and adverse outcomes^1^^-^ 3. Mineral and bone disorders (MBD) are major complications of CKD, resulting in several clinical consequences, such as fractures, bone pain, skeletal deformities, vascular calcification, cardiovascular disease, and death^3^^,^ 4.

Much progress has been achieved toward understanding CKD-MBD pathophysiology over the last decades^5^^-^ 10. In contrast, the consequences in clinical outcomes related to distinct histological patterns of renal osteodystrophy (RO) are largely unknown. This problem becomes more complex considering RO-associated diagnoses, such as low trabecular bone volume (osteoporosis) and metal accumulation.

The available information regarding RO has mainly been derived from a series of observational studies showing a trend in the prevalence of high turnover disease, a decrease in osteomalacia, and stabilization of adynamic bone disease over the last decades^11^^-^ 15. A recent study observed a modification in the proportion of RO distribution, suggesting that low bone turnover disease can occur frequently during the late stages of CKD^16^. None of these studies prospectively addressed the clinical outcomes according to the type of RO, presence of osteoporosis, or aluminum accumulation.

The European Renal Osteodystrophy initiative was created in 2016 to revitalize bone biopsy as a clinically useful tool 17 ^,^ 18. Two years earlier, the Brazilian Registry of Bone Biopsy (REBRABO) was launched as a national and prospective registry serving as a research platform to expand knowledge regarding CKD-MBD.

REBRABO is an electronic database that contains clinical, laboratory, imaging, and histological data of CKD-MBD patients who underwent bone biopsy in Brazil, and it could contribute to a better knowledge regarding the clinical consequences of RO. Furthermore, data analyses based on REBRABO could provide updated epidemiological and clinical information related to RO and CKD-MBD in Brazil^19^^,^ 20.

The present study is the first prospective analysis of REBRABO data at the 30-month time-point. We aimed to describe the epidemiological profile of RO in a sample of CKD-MBD Brazilian patients and understand the relationship between RO and associated diagnoses and outcomes.

## MATERIALS AND METHODS

### STUDY DESIGN

This paper presents a national, multicenter, observational and prospective study. The selection criteria were as follows: CKD-MBD patients in stages 3-5D according to the Kidney Disease Improving Global Outcomes (KDIGO)^21^ guideline who underwent bone biopsy as indicated by a Nephrologist and had clinical, laboratorial, and imaging data inputted into the REBRABO system between August 2015 and March 2018. Bone biopsy had one of the following indications: persistent bone pain, unexplained hypercalcemia/phosphatemia, nontraumatic bone fractures, prebisphosphonate therapy or preparathyroidectomy, suspicion of aluminum (Al) accumulation, and research protocol. The exclusion criteria were as follows: refusal to sign the informed consent form, age <18 years, estimated creatinine clearance ≥ 60 mL/min, current kidney transplantation, and bone biopsy indicated by a specialty other than nephrology.

**Figure 1:**
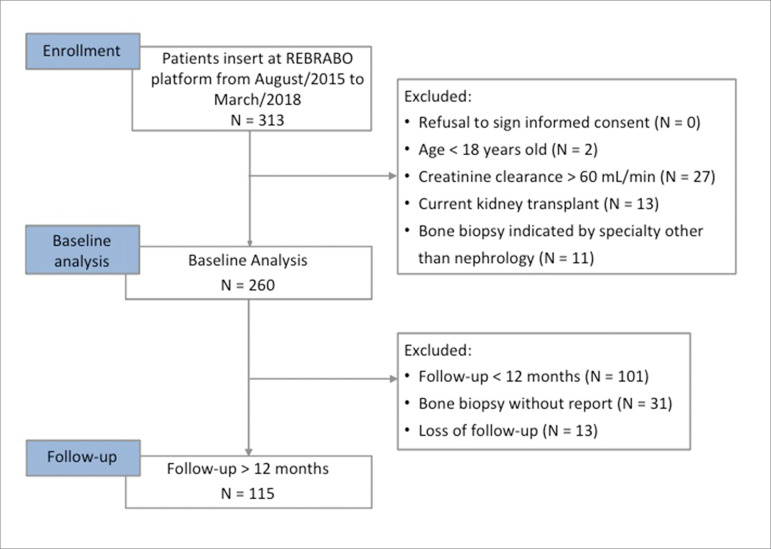
Study design.

Baseline was defined as the time when the patient underwent bone biopsy. The prospective analysis included data from patients who completed at least 12 months of follow-up. Patients who could not be located or stopped their treatment and patients whose biopsies had not been analyzed until March 2018 were excluded from the prospective analysis (Figure 1). Follow-up continued until 30 months after the study began.

This study was approved by the Ethical and Research Committee of UNICAMP (CAAE: 4131141.6.0000.5404) and performed according to the guidelines of the Declaration of Helsinki. Informed consent was obtained from all patients or their caregivers.

### CLINICAL, DEMOGRAPHIC, LABORATORY AND IMAGING DATA

The clinical, demographic, and laboratory data were collected at baseline and follow-up using standard electronic forms available at the REBRABO web system. The baseline data were entered by a Nephrologist who performed the bone biopsy and checked by a single researcher. The follow-up data were obtained by telephone from the Nephrologist who performed the bone biopsy, the dialysis unit, and the patients. The included data were always entered by the same researcher and checked by another researcher. Patients with a history of coronary disease, stroke, and obstructive peripheral arterial disease were considering to have experienced previous major adverse cardiovascular events (MACE).

To reduce variation between laboratories in the serum parathormone (PTH) and total alkaline phosphatase levels, REBRABO requests information regarding the reference ranges of these parameters. Based on KDIGO recommendations, the following 3 categories of serum PTH levels were created considering the upper limit of the reference range: “within target (PTH between 2-9x)”; “below target (PTH < 2x)”; and “above target (PTH > 9x)”^4^. The serum total alkaline phosphatase levels were classified as “below”, “within”, or “above” the reference from the local laboratory. Hyperphosphatemia was considered if the serum phosphate levels were ≥ 4.7 mg/dL. An analogue visual pain scale was used to measure the referred intensity of “bone pain”. Bone pain was categorized as moderate to severe if the patient provided a score ≥ 3.

## BONE TISSUE ANALYSIS

Bone fragments were obtained via transiliac bone biopsies and undercalcified bone fragments were submitted to standard processing for histological studies under toluidine blue staining. Tetracycline double labeling was used, and the histological analysis was performed by a single researcher. The samples from individual patients were classified as having osteitis fibrosa, mixed uremic osteodystrophy, adynamic bone disease, or osteomalacia according to their respective TMV status. The presence of low trabecular bone volume and Al accumulation were considered indicative of an RO-associated diagnosis^7^^,^ 8. For the purpose of equivalence and clinical translation, we assumed the term “low bone volume” to indicate “osteoporosis”, based on the observations of Malluche *et al.* 22. Al accumulation was considered if more than 30% of the surface of the trabecular bone was covered by this metal under solochrome-azurine staining. To discriminate Al accumulation from iron accumulation in bone, Perls’ staining was performed.

### DEFINITION OF OUTCOMES

Nontraumatic bone fractures, hospitalization, and death were considered the primary outcomes.

### STATISTICAL ANALYSIS

The continuous variables are reported as the means ± SD or medians/interquartile intervals. The categorical data are reported as frequencies/percentages. Comparisons between the continuous variables, skewed data, and categorical variables were performed using a Student’s t-test, Mann-Whitney test, and X^2^ test, respectively. The McNemar test, Student’s t-test, and Wilcoxon test were used to evaluate the differences between the baseline and follow-up data. A multivariate stepwise linear regression and logistic regression were performed to identify the independent determinants of “osteoporosis” and “Al accumulation”. Variables selected from the univariate regression (dialysis vintage and modality, serum PTH, alkaline phosphatase, 25-hydroxyvitamin D, bone turnover, Al accumulation and previous parathyroidectomy) were included in the multivariate model of “osteoporosis”. Variables selected from the univariate regression (age, gender, estimated duration of CKD, dialysis vintage and modality, body mass index, serum PTH, alkaline phosphatase, previous parathyroidectomy, and kidney residual function) were included in the multivariate model of “Al accumulation”. A COX regression analysis of the determinants of “hospitalization” and “death” was performed. The variables selected for “hospitalization” included age, diabetes, MACE, diagnosis of osteoporosis, and serum hemoglobin. The variables selected for “death” included age, diabetes, MACE, dialysis vintage, diagnosis of mixed uremic osteodystrophy, and total serum calcium. Statistical analyses were performed using SPSS 22.0 (SPSS Inc., Chicago, IL). A two-sided *p*-value < 0.05 was considered statistically significant.

## RESULTS

### GENERAL CHARACTERISTICS OF THE POPULATION AT BASELINE

Between August 2015 and March 2018, data from 313 patients were available at the REBRABO system; 260 patients were included according to the study criteria at baseline. Table 1 describes the clinical and biochemical findings. The patients presented a high prevalence of symptoms and signs related to quality of life and abnormalities in the serum biochemistry.

**Table 1:** Clinical and serum biochemical findings at baseline

Parameter	N = 260
Age (years)	51 ± 12
Gender (male; N, %)	133 (51)
Ethnicity (Caucasian; N, %)	105 (40)
Body mass index (kg/m^2^)	24 (22 - 27)
Cause of CKD (N, %)	
Hypertension	79 (30)
Chronic glomerulonephritis	48 (19)
Diabetes mellitus	30 (12)
Estimated duration of CKD (months)	115 (39 - 202)
Conservative management (N, %)	24 (9)
Modality of dialysis (hemodialysis; N, %)	211 (89)
Dialysis vintage (months)	86 (39 - 168)
Previous parathyroidectomy (N, %)	56 (22)
Bone fractures (nontraumatic)	40 (15)
Symptoms and signs (N, %)	
Bone pain / moderate or severe	135 (54) / 84 (33)
Weakness	110 (42)
Myalgia	78 (30)
Bone deformity	45 (17)
Pruritus	38 (15)
Tendon rupture	10 (4)
Serum biochemistry*	
Albumin (g/dL)	3,9 (3,5 - 4,2)
Creatinine (mg/dL)	8,9 (6,6 - 10,6)
Hemoglobin (g/dL)	11,5 (10,3 - 13)
Total calcium (mg/dL)	9,3 (8,6 - 9,8)
Phosphate (mg/dL)	5 (4 - 6,2)
Alkaline phosphatase (IU/L)	110 (75 - 204)
Parathormone (pg/mL)	217 (47 - 636)
25-OH vitamin D (ng/dL)	28 (21 - 36)
Aluminum (µg/L)	9,35 (4,75 - 19,25)

CKD, chronic kidney disease;

* N of samples = 46 for serum aluminum levels, 107 for 25-OH vitamin D levels, and 155 for albumin levels.

The serum PTH levels were within the range recommended by KDIGO in 88 (34%) patients. The serum alkaline phosphatase and 25-OH vitamin D levels were within the recommended range in 162 (62%) and 48 (45%) patients, respectively; hyperphosphatemia was observed in 142 (55%) patients, while hypercalcemia was observed in 32 (13%) patients.

The main indications for bone biopsy were as follows: research protocol in 107 (41%) patients, suspected aluminum accumulation in 80 (31%) patients, persistent bone pain in 34 (13%) patients, unexplained hypercalcemia/phosphatemia in 14 (5.4%) patients, nontraumatic bone fractures in 11 (4.2%) patients, prebisphosphonate therapy in 8 (3.1%) patients, and preparathyroidectomy in 6 (2.3%) patients.

### RENAL OSTEODYSTROPHY AND ASSOCIATED DIAGNOSES

**Figure 2:**
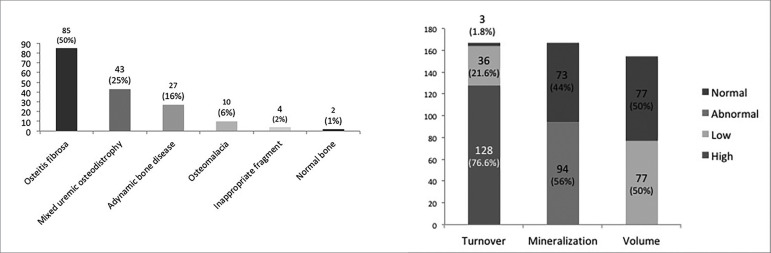
Distribution pattern of renal osteodystrophy and turnover-mineralization-volume (TMV) classification (N = 173).

Of the 260 patients included at baseline, 171 (66%) patients had reports of an RO diagnosis in March 2018. Osteitis fibrosa and mixed uremic osteodystrophy were the most prevalent forms of RO and were detected in 85 (50%) and 43 (25%) patients, respectively. The distribution pattern of RO and the TMV classification are shown in Figure 2. There were no significant changes in the distribution of RO patterns or in the TMV classification when considering data from patients whose indication of bone biopsy was based on a research protocol.

The relationship between the TMV classification and the symptoms/signs was unspecific, except among patients diagnosed with osteoporosis, who experienced a higher prevalence of moderate-severe bone pain than patients with a normal bone trabecular volume [41 (54%) *vs*. 20 (27%); *p =* 0.001)], and patients diagnosed with abnormal mineralization, who presented a higher prevalence of myalgia than patients with normal mineralization [37 (39%) *vs*. 17 (22%); *p =* 0.03)].

Osteoporosis was diagnosed in 77 (44%) patients. There was no significant difference in the prevalence of osteoporosis among the patients with different types of RO [37 (46%) cases of osteitis fibrosa, 19 (51%) cases of mixed uremic osteodystrophy, 4 (50%) cases of osteomalacia, and 16 (67%) cases of adynamic bone disease (*p =* 0.35)]. The logistic regression showed that dialysis vintage was an independent predictor of osteoporosis (OR: 1.005; CI: 1.001-1.010; *p =* 0.01).

Al accumulation on the trabecular bone surface was detected in 65 (38%) patients. There was no significant difference in the prevalence of Al accumulation among the patients with different types of RO [32 (38%) cases of osteitis fibrosa, 16 (37%) cases of mixed uremic osteodystrophy, 4 (40%) cases of osteomalacia, and 10 (38%) cases of adynamic bone disease (*p =* 0.9)]. The same findings were observed according to the TMV classification [14 (39%) cases of low bone turnover *vs*. 48 (37%) cases of high bone turnover (*p =* 0.4); 24 (33%) cases of normal mineralization *vs*. 38 (40%) cases of abnormal mineralization (*p =* 0.31); and 30 (39%) cases of normal trabecular bone volume *vs*. 26 (34%) cases of low trabecular bone volume (*p =* 0.54)]. The multivariate logistic regression revealed the following independent predictors of bone Al accumulation: hemodialysis treatment (OR: 11.24; CI: 1.227-100; *p =* 0.03), previous parathyroidectomy (OR: 4.97; CI: 1.422-17.241; *p =* 0.01), and female gender (OR: 2.88; CI: 1.080-7.679; *p =* 0.03).

The diagnosis of Al accumulation in 33 (51%) patients by the nephrologists was an unexpected finding. Even in patients who underwent bone biopsy indicated based on a research protocol, the prevalence of Al accumulation reached similar rates [65 (38%) *vs*. 58 (47%), *p =* 0.1]. Overall, the suspicion of bone Al accumulation by a nephrologist represented a clinical test with low sensitivity (54%; positive predictive value of 46%), and low specificity (65%; negative predictive value of 32%).

### FOLLOW-UP STUDIES

The mean follow-up duration was 21±5 months, and 115 (44%) patients were included in the prospective analysis. No significant change in the prevalence of signs/symptoms was detected throughout the follow-up period. There was a trend toward a reduction in serum phosphate levels [5.0 (3.9 - 6.5) to 4.5 (3.5 - 5.4) mg/dL (*p =* 0.07)] and a significative change in serum alkaline phosphatase levels [116 (71 - 210) to 108 (67 - 278) (*p =* 0.01)].

Seven (7%) patients had an episode of nontraumatic fracture at follow-up as follows: 2 (29%) fractures of femur, 2 (29%) fractures in toes, 1 (14%) fracture in ribs, 1 (14%) fracture in clavicle, and 1 (14%) fracture in an undetermined site. These patients were prone to have diagnosis of diabetes [2 (40%) *vs*. 5(5%), *p =* 0.04] and had a history of MACE [3 (30%) *vs*. 4 (5%), *p =* 0.023].

**Figure 3:**
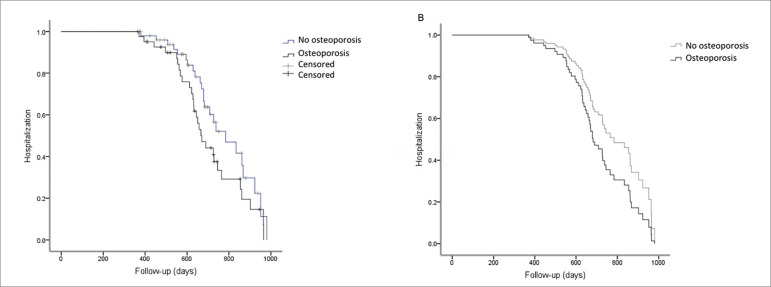
Kaplan-Meier and Cox regression analyses of survival in the outcome “hospitalization” according to the diagnosis of osteoporosis.(A) Kaplan-Meier curve unadjusted for the outcome “hospitalization” according to the diagnosis of osteoporosis [p (log-rank) = 0.08]; (B) Cox survival curve adjusted for age, diabetes, MACE, serum hemoglobin level and osteoporosis. There was no significant difference in the model (*p =* 0.152) (N = 93; “hospitalization”-censored was noted in 40 patients).

There were 56 hospitalizations, including 15 (27%) due to cardiovascular disease. These patients were prone to have low serum hemoglobin levels (11.1 ± 2 *vs*. 12.1 ± 2 g/dL, *p =* 0.01) and a diagnosis of osteoporosis [29 (69%) *vs*. 24 (46%), *p =* 0.03]. The Kaplan-Meier curve analysis showed a trend for “hospitalization” between patients with (N = 42) and without (N = 52) osteoporosis [29 (69%) *vs*. 24 (46%) events; p (log-rank) = 0.08]. However, the COX regression analysis revealed that none of the variables was an independent predictor of “hospitalization” (*p =* 0.152) (Figure 3).

**Figure 4:**
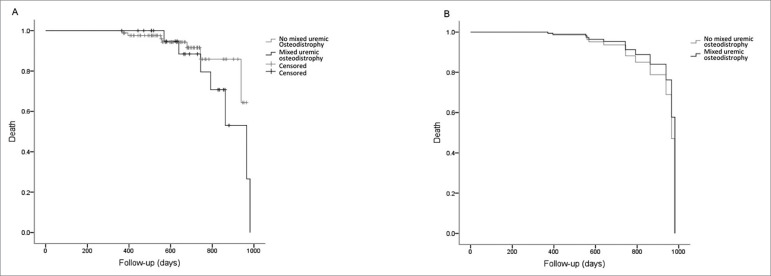
Kaplan-Meier and Cox regression analyses of survival in the outcome “death” according to the diagnosis of mixed uremic osteodystrophy. (A) Kaplan-Meier curve unadjusted for the outcome “death” according to a diagnosis of mixed uremic osteodystrophy [p (log-rank) = 0.4]; (B) Cox survival curve adjusted for age, diabetes, MACE, dialysis vintage, a diagnosis of mixed uremic osteodystrophy, and serum total calcium levels. There was no significant difference in the model (*p* = 0.34) (N = 97; “death”-censored was noted in 13 patients).

Fourteen (12.5%) deaths were detected at follow-up, 6 (38%) due to cardiovascular disease. These patients were prone to be older (58 ± 10 *vs*. 51 ± 11, *p =* 0.03) and have more dialysis vintage (163 ± 108 *vs*. 99 ± 84, *p =* 0.014), diagnosis of mixed uremic osteodystrophy [7 (28%) *vs*. 7 (8%), *p =* 0.009], low total serum calcium levels (8.7 ± 0.8 *vs*. 9.3 ± 0.9, *p =* 0.038), calcium carbonate use [6 (26%) *vs*. 8 (9%), *p =* 0.027], and sevelamer use [4 (40%) *vs*. 10 (10%), *p =* 0.006]. Although more deaths occurred among patients with Al intoxication (7 in 35 patients) compared to those without (7 in 77 patients), no statistical significance was noted (*p =* 0.1). The COX regression revealed that none of the variables was an independent predictor of death (*p =* 0.34) (Figure 4).

## DISCUSSION

This study presents the first prospective analysis of REBRABO data at the 30-month time-point and reveals the following main results: first, the patients had a high prevalence of clinical symptoms, and the relationship between symptoms and the type of RO was unspecific; second, high bone turnover diseases were more prevalent; third, a high prevalence of osteoporosis and Al accumulation was detected but was not predictive of the outcomes; and finally, RO or TMV classification was not related to hospitalization and death outcomes.

Clinical symptoms related to CKD-MBD are difficult to interpret because they are nonspecific, subjective, and frequent in CKD patients^23^^,^ 24. In addition to highlighting the high prevalence of unexpected diagnoses revealed only by bone biopsies, our results underline the difficulty faced by nephrologists in predicting diagnosis and guiding treatment for RO based on clinical manifestations.

The high prevalence of moderate to severe bone pain in patients with osteoporosis is apparently conflicting. However, we must keep in mind that pain is multifactorial in CKD patients, and may be influenced by factors such as uremic neuropathy, diabetic neuropathy, peripheral arterial insufficiency, amyloidosis, among other factors 25. Some patients with diagnosis of osteoporosis also had fibrous osteitis, with high serum PTH levels, which may explain at least partially, the occurrence of bone pain in patients with the diagnosis of osteoporosis.

It was not part of the scope of the study to evaluate in a controlled manner the influence of bone biopsy findings on therapeutic decisions or quality of life. However, we believe that, for specifics groups of patients, results from bone biopsy were able to improve their symptoms or clinical condition, as in the cases of Al accumulation or to corroborate the indication of surgical parathyroidectomy.

Information regarding the actual prevalence of different types of RO is conflicting^14^^-^ 16. These discrepancies could be attributed to many factors, such as differences in the epidemiological and clinical characteristics of the CKD patients (such as age or ethnicity), availability of drugs used for the treatment of CKD-MBD, and treatment modalities among the populations studied^11^^-^ 16 ^,^ 26 ^,^ 27. However, whether a specific histological type of renal RO could result in unfavorable clinical outcomes and whether it would only mark the severity of a broader disease dissociated from outcomes is unknown.

Some evidence suggests that a relationship exists between adynamic bone disease or very low serum PTH levels and high mortality, but the interpretation of this information is biased by many other factors, such as vascular calcification, fractures, and hormonal changes 28 ^,^ 29. In contrast, extremely high serum PTH levels as a marker of high turnover bone disease are also linked to high mortality^30^. To the best of our knowledge, our study is the first to show that neither the distinct patterns of RO nor the TMV characteristics impact the outcomes at least at this relatively short-term time-point.

We detected a high prevalence of Al accumulation in bone tissue. These results contradict previous findings in the scientific literature that support that Al accumulation in bone has no epidemiological relevance^31^^-^ 33. However, some of these studies are considered old and mainly based on observations of nontoxic concentrations of Al in blood from CKD patients and in hemodialysis water^32^^,^ 33. Based on our findings, we speculate whether this problem is regional or occurs in many other countries in a silent or underdiagnosed way.

Interestingly, the presence of Al deposition in bone was not related to the type of RO or TMV classification. Other factors, such as female gender, were associated to a 3-fold higher risk for Al accumulation, and prior parathyroidectomy increased the risk by 5 folds. The reasons underlying these associations are unknown and probably involve Al kinetics, diffusional clearance, and hormonal factors (estrogen and PTH)^34^. Water used for hemodialysis, intestinal absorption, medications, and parenteral and polyelectrolyte concentrate solutions remain possible sources of Al^35^^-^ 38.

It is estimated that CKD patients have a 4 times higher risk of femur fracture than the general population due to several factors, including high prevalence of osteoporosis, changes in biochemical and endocrine parameters, and uremic toxins^39^^-^ 41. Since we detected a low number of cases of bone fractures in our study, it was not possible to assess in depth the eventual relationships with the clinical, biochemical, and histological parameters.

Our study has limitations. The laboratory tests were not standardized, and a considerable proportion of patients had no data available at follow-up time-point. The thirty-month follow-up period was possibly too short to observe outcomes, such as bone fractures and death; 13 (5%) patients were removed during the follow-up period due to the impossibility of contacting them or non-registered death. Another limitation is the lack of full histomorphometric studies, which makes it impossible to clarify, for example, why some patients with the diagnosis of osteomalacia (which should present as having high bone volume) had the associated diagnosis of osteoporosis. However, the intrinsic characteristic of being a real-life study, the inclusion of many patients and the prospective analysis of important outcomes are strengths of this study. Although the diagnoses of low trabecular bone volume, mixed uremic osteodystrophy, dialysis vintage, serum hemoglobin, and total calcium presented some relationships with hospitalization or death, none of these parameters could be identified as independent factors linked to outcomes.

In conclusion, this prospective analysis of REBRABO data at the 30-month time-point revealed that hospitalization and death were not influenced by the different histological patterns of RO or the TMV classification. An elevated prevalence of high turnover diseases, osteoporosis, and Al accumulation was observed in our population. As the diagnosis of Al accumulation was performed at the subclinical level in most patients, we suggest that new studies including bone biopsy be performed to detect the real prevalence of this condition in different regions worldwide.
